# Revised time estimation of the ancestral human chromosome 2 fusion

**DOI:** 10.1186/s12864-022-08828-7

**Published:** 2022-08-25

**Authors:** Barbara Poszewiecka, Krzysztof Gogolewski, Paweł Stankiewicz, Anna Gambin

**Affiliations:** 1 Institute of Informatics, Warsaw University, Warsaw, Poland; 2 Department of Molecular and Human Genetics, Baylor College of Medicine, Houston, TX US

**Keywords:** Human chromosome 2, Chromosomal fusion, Biased gene conversion, Inclusion-exclusion principle, Confidence interval, Speciation

## Abstract

**Background:**

The reduction of the chromosome number from 48 in the Great Apes to 46 in modern humans is thought to result from the end-to-end fusion of two ancestral non-human primate chromosomes forming the human chromosome 2 (HSA2). Genomic signatures of this event are the presence of inverted telomeric repeats at the HSA2 fusion site and a block of degenerate satellite sequences that mark the remnants of the ancestral centromere. It has been estimated that this fusion arose up to 4.5 million years ago (Mya).

**Results:**

We have developed an enhanced algorithm for the detection and efficient counting of the locally over-represented weak-to-strong (AT to GC) substitutions. By analyzing the enrichment of these substitutions around the fusion site of HSA2 we estimated its formation time at 0.9 Mya with a 95% confidence interval of 0.4-1.5 Mya. Additionally, based on the statistics derived from our algorithm, we have reconstructed the evolutionary distances among the Great Apes (*Hominoidea*).

**Conclusions:**

Our results shed light on the HSA2 fusion formation and provide a novel computational alternative for the estimation of the speciation chronology.

## Introduction

The ancestral chromosomal fusion, creating the human chromosome 2 (HSA2) and reduction of the chromosome number from 48 in the Great Apes to 46 in modern humans was described nearly four decades ago [1–3]. To better understand this event, the 2q13-q14.1 fusion site has been analyzed using different computational and molecular methods.

### Current knowledge about the HSA2 fusion site

Fluorescence in-situ hybridization (FISH) analyses confirmed that two ancestral Great Ape chromosomes fused at their telomeric repeats to form the HSA2 [4]. Subsequent studies confirmed also the presence of multiple subtelomeric segmental duplications (SD) homologous to other autosomal chromosomes [5] and described the gene content at the fusion site [6, 7]. Additionally, the comparison of SDs between the chimpanzee and human genomes not only enabled estimation of the genomic duplication rate, but also suggested SDs as the key cause of transcriptional differences between species and the formation of the ancestral fusion. A 40 kb SD near the fusion site has been identified in 300-500 copies in the chimpanzee genome but only in 4-5 copies in the modern human genome [8].

Using the yeast genome with the functional single-chromosome as a model, it was shown that the reduction of the number of chromosomes does not always have to lead to fatal genetic dysfunctions [9, 10].

### When did the fusion event occur? Time estimation approaches

These genomic observations have raised questions about the time scope when this gross chromosomal aberration arose. Dreszer et al. [11] have proposed a time estimation method based on the analysis of the fixed substitutions in the human and chimpanzee genomes since their divergence from the common ancestor. The authors have referred to the biased gene conversions (BGCs) occurring due to the mutagenic recombination events [12] and the associated DNA repair processes to favor strong (GC) versus weak (AT) nucleotide pairs at the non-Watson-Crick heterozygous sites in heteroduplex DNA [13]. Importantly, it has been broadly discussed that BGC may be one of the main evolutionary mechanism [14, 15]. However, Dreszer et al. observed that particularly weak-to-strong (AT to GC) substitutions over-represented locally, e.g. clustering densely near the telomeres of the autosomal chromosomes. Furthermore, using the Unexpected Bias Clustered Substitutions (UBCS) statistics measuring the bias towards weak-to-strong substitutions among the clustered substitutions, a similar over-representation for human and chimpanzee orthologous regions was detected. This observation suggested the existence of a stable evolutionary force that had led to the formation of the biased clusters of substitutions. As expected, around the ancestral HSA2 fusion site, an additional local maximum of the UBCS statistic values was determined. To approximate the time of the fusion event Dreszer et al. assumed that: (i) human-chimpanzee split had occurred 6 Mya and (ii) the rate of the UBCS accumulation is constant. Based on that, they compared the reduction of the bias in the regions near the fusion site with the orthologous telomeric sites of the chimpanzee chromosomes 2A and 2B. As a result, they estimated the fusion time at 0.74 Mya with a 95% confidence interval 0-2.81 Mya.

A phylogenetic analysis of the SVA elements (i.e. composite repetitive elements named after its main components, SINEs, VNTRs and *Alu*s) was performed by Wang et al. [16]. The authors showed that within this hominid-specific retroposone family, both SVA-E and the SVA-F subfamilies are restricted to the human lineage. Additionally, based upon the nucleotide divergence, they estimated the expansion time of these subfamilies at 3.5 Mya (with a GC content-dependent range of 2.5-4.5 Mya), which provided a lower bound of the human-chimpanzee speciation event.

In support of these estimations, using the next generation sequencing (NGS) with a high read coverage, Meyer et al. [17] have reconstructed a genome of the *Denisovans*, an extinct relative of the *Neandertals*, and identified an evidence of the HSA2 fusion event. These findings corroborated the theory that the *Denisovans* (and presumably also the *Neandertals*) had shared the fused HSA2 with modern humans. Moreover, the studies on the shared centromere sequence organization in the *Denisovan* and *Neandertal* genomes provided an additional premise that the HSA2 fusion arose prior to our last common ancestor with *Hominins* [18].

### Our results

We present the revised estimation of the HSA2 fusion time. Our results are twofold. First, we developed a novel algorithm for the re-calculation of the UBCS statistics defined by Dreszer et al. [11]. The estimation procedure of the expected number of the so-called clustered substitutions was modified through the introduction of the inclusion-exclusion principle. Our approach allows to calculate the exact value of UBCS statistic even for the complex structures of the intersecting clusters, which was unattainable with the original method. Consequently, we calculated the UBCS statistics for the Great Apes family and the updated estimation of the HSA2 fusion time. Furthermore, we discuss how the UBCS statistics can be used to derive evolutionary distances within the Great Apes family. Finally, we present an observation on the linearity of the number of biased clustered substitutions (BCS) occurrences with respect to time.

In the following section, we introduce the genomic datasets used in this study, i.e. the Great Apes, and modern humans. We then describe in detail the UBCS statistics and discuss its deficiencies and potential oversights. Next, we comment on the introduced changes in the UBCS statistics and their impact on the estimation of the ancestral fusion time. We point out other observations regarding the evolutionary events related to weak-to-strong mutations. Finally, we discuss the possible improvements that could be implemented into our analyses, especially when the missing fragments of the Great Apes chromosomes are available.

## Results

**Fig. 1:**
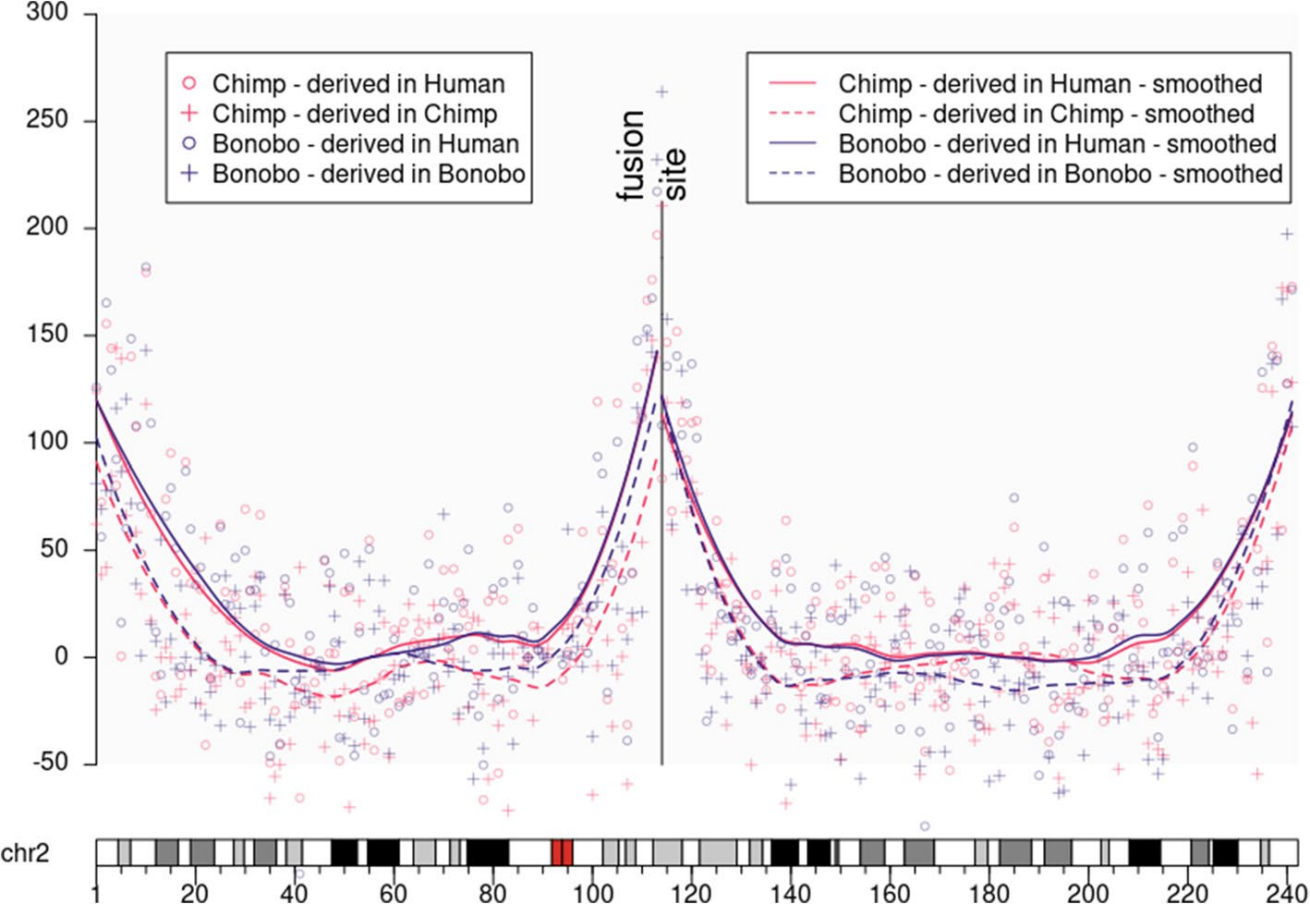
UBCS statistic for human chromosome 2. The figure above presents the values of the UBCS statistics along the whole chromosome 2. The vertical line denotes the ancestral fusion site point (chr2:113,500,000). One can observe how the lines corresponding to the same organism (solid vs dashed) differ from each other settling the difference between time estimation of the ancestral evolutionary split

We present a revised estimation of the ancestral HSA2 fusion date based on the modified UBCS statistics. Furthermore, we present how the statistics corresponds to the evolutionary distances between human and Great Apes. Using the UBCS proportion between species, we have calculated the rates in which the BCS occurred in the telomeric regions. We have then used them to predict the timeline of the evolutionary events in the human lineage.

### Revised HSA2 fusion date

First, after Dreszer et al. [11], we have applied the UBCS statistics using the single nucleotide differences with a region of orthology in chimpanzee (*Pan troglodytes*). Additionally, we have added its evolutionary relative bonobo (*Pan paniscus*) to verify whether the UBCS statistics are consistent as might be expected in the context of evolutionary research [19].

In Fig. 1, we present the UBCS statistics values for both species that clearly indicate the HSA2 fusion site. Consequently, we have re-estimated the ancestral fusion date using the comparisons between the chimpanzee and modern human genomes to approximately 0.9 Mya with a 95% confidence interval of 0.4 - 1.5 Mya.

**Fig. 2:**
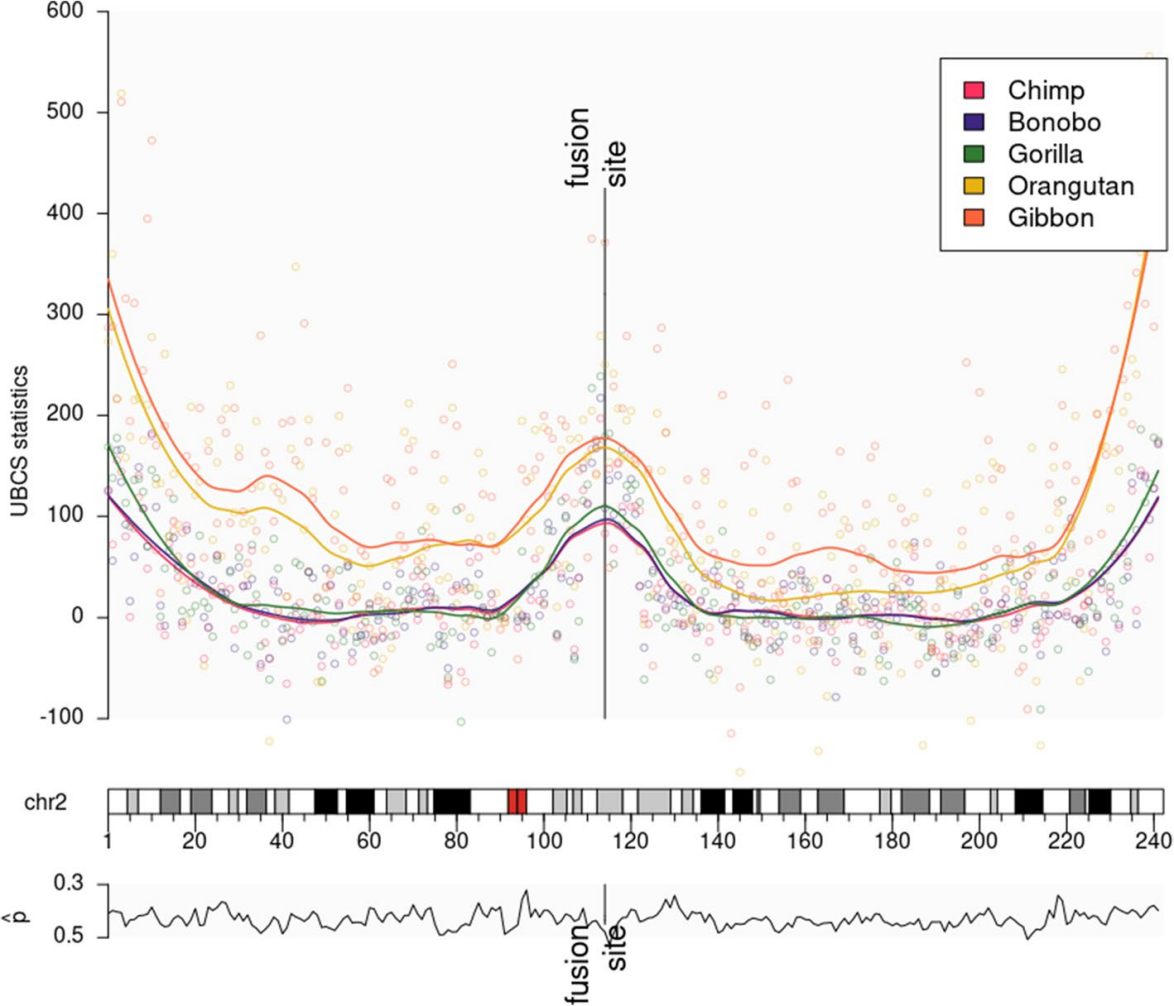
UBCS statistic for human chromosome 2 and Great Ape genomes. (Top panel) UBCS statistic for substitutions derived in human are depicted as dots. Lines are the UBSC statistic values smoothed using loess regression. HSA2 shows the peak of the UBCS values near the ancestral fusion site. Atypical central peak occurs for the UBCS statistic computed using comparisons of the human to all Great Ape genomes. (Bottom panel) Values of $\hat{p}$ parameter (proportion of weak-to-strong in all substitutions) for every 1 Mb window of substitution derived in human chromosome 2

Additionally, we have applied the same procedure of the fusion time estimation to the pair of the bonobo (*Pan paniscus*) and the modern human genomes. Since currently it is assumed that the present-day bonobo species have diverged from the common ancestor with modern human at the same time as chimpanzee [19], we expected that the estimation of the HSA2 fusion time will be similar to the one calculated based on the chimpanzee genome. Nonetheless, a time point was estimated as 0.67 Mya with 95% confidence interval 0-1.3 Mya. On one hand, this result contradicts the evolutionary reports. On the other hand, we observed a clear difference between the mutational dynamics of BCS on both sides of the fusion site. The proximal side maintains full compatibility between species, while on the distal side there is a double difference between species. In the next chapter, we discuss the possible reasons of these differences.

### Coincidence of UBCS and evolutionary distances among Great Apes

Similarly as above, we have applied the UBCS statistics using single nucleotide differences within a region of homology to three more * hominidae* species: gorilla (*Gorilla Gorilla*), orangutan (*Pongo pygmaeus abelii*), and gibbon (*Nomascus leucogenys*). We show that for all five species, the UBCS statistics around the fusion site is monotonic as a measure of evolutionary distance (i.e. that species that are more evolutionary distant from human, have speciated prior to the others that have higher values of this statistics, see Fig. 2).

**Fig. 3:**
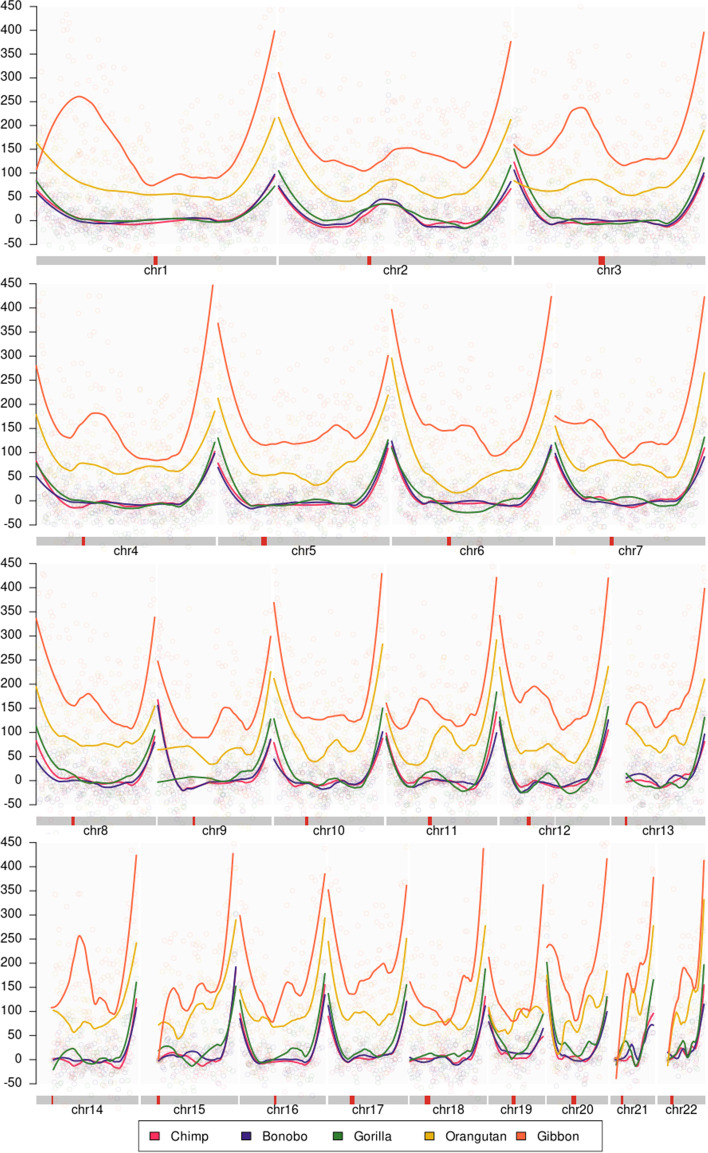
UBCS statistic for all autosomal chromosomes. The figure presents the value of the UBCS statistics over autosomal chromosomes for all five Great Apes studied in this paper

Furthermore, based on the observation that the telomeric values of the UBCS statistics are consistently elevated for all autosomal chromosomes among all Great Apes (see Fig. 3), we have searched for the irregularity pattern. We have studied the relationship between the enrichment of BCS, thus values of the UBCS statistics, and evolutionary distances between organisms.

**Fig. 4:**
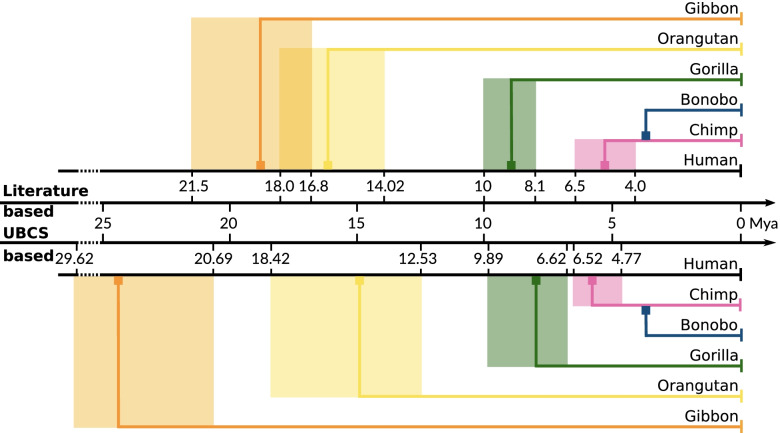
Evolutionary distances between Great Apes and Human. All recent reports about the possible speciation events times are shown. For each species, the minimal and the maximal dates are denoted on the horizontal time axis. Using the UBCS statistics proportions, we have estimated the time of the following divergence events from the human lineage for all species: Chimpanzee: 4.77-6.52 Mya, Bonobo: 4.35-5.85 Mya, Gorilla: 6.62-9.89 Mya, Orangutan: 12.53-18.42 Mya, Gibbon: 20.68-29.62 Mya. Please note, that each period calculated with the timeframe overlaps the with time frames taken from the literature

In the literature there are many reports aiming to estimate the speciation date of *hominidae* species from human (see Fig. 4). Starting chronologically, using Bayesian analysis with the relaxed clock model, the last common ancestor (LCA) of Gibbon *(Nomascus leucogenys)* and human was estimated by Chan et al. [20] to have lived 19.25 Mya (95% confidence interval: 15.54-22.99 Mya). Using the relaxed clock model Chatterjee et al. estimated this event at 21.5 Mya (18.9-24.3) [21]. Carbon et al. [22] suggested $\tilde{1}6.8$ Mya (15.9-17.6) assuming a split time with macaque of 29 Mya and using the Bayesian coalescent-based methodology [23]. Next, Orangutan *(Pongo Pygmaeus Abelli)* was estimated to speciate 18 Mya [24] by applying the maximum likelihood (ML) method to intron sequences of 20 different loci. Later, a split time of 14.02 Mya (12.24-15.89) was suggested by Chan et al. using the same method as for gibbons [20]. Chatterjee provided an estimation of 15.9 Mya (13.7-18.3). Speciation of Gorilla *(Gorilla Gorilla)* population by Chan et al. [20] was estimated at 8.95 Mya (6.95-11.08). Raaum et al. [25] suggested 8.1 Mya (7.1-9.0), whereas Scally et al. [26] based on assembly and analysis of a genome sequence and fossil evidence places the specialization event at approximately 10 Mya. Further, based on coalescent hidden Markov model framework using in the context of incomplete lineage sorting, the existence of the LCA of chimpanzee and human was estimated at 6 Mya by Scally et al. [26], 4 Mya by Hobolth et al. [27] and 6.5-4.2 Mya by Stone et al. [28] (see also references therein).

Based on the cited literature reports describing the estimations of the LCAs between various species and the over-representation of BCS near telomeric regions, we have found a specific relationship between UBCS statistics proportion and evolutionary distances for two given species. Using procedure described in the Methods section, we have calculated the speciation time for each pair of species based on their UBCS proportion $\mathcal {G}_{x} \vert \vert \mathcal {G}_{y}$. For each species, for both minimal and maximal speciation time from the literature, we have estimated the average speciation value with respect to other species. As a result, we report the predictions of the speciation dates for all successive species. Chimpanzee and bonobo were are estimated to diverge very close to each other, between 4.7-6.6 Mya and 5.5-7.5 Mya, respectively. For gorilla, orangutan, and gibbon, the estimates are, respectively, 6.6-9.9 Mya, 12.5-18.4 Mya, 20.7-29.6 Mya. Overall, our results are consistent with the literature reports; however, the elder two species have a bit less robust estimation (see Fig. 4). In the Section 4, we comment on the quality of this estimation as well as possible future improvements.

## Discussion

Here, we provide a revised method for calculation of the UBCS statistics proposed by Dreszer et al. [11]. We have re-calculated the time of the HSA2 fusion event at approximately 0.9 Mya (0.4-1.5 Mya), using the same human and chimpanzee genomes comparison [11]. To verify our approach, we have used the bonobo genome as query because of their common evolutionary history [19]. Interestingly, our results suggest that the fusion might have occurred more recently, approximately 0.6 Mya. We propose that this discrepancy may result from the quality of the bonobo genome assembly. Using the UCSC Browser [29], we have observed that the genomic region distal to the HSA2 fusion site maps well to the near-telomeric region of chimpanzee chromosome 2B, and thus the corresponding UBCS statistics has high values (Fig. 1). ). Conversely, the genomic region proximal to the fusion site maps to the ambiguous region surrounded by closely located centromere and the large sequence gap. This observations may explain that the HSA2 fusion had a head-to-head type, but likely a big telomeric and sub-telomeric portion containing genes was lost. [30].

Furthermore, we draw the reader’s attention to the speciation estimation among the Great Apes. The short literature review described in the previous section presents how imprecise these estimations are. The differences in the calculated dates of the speciation events span from 2.5 Mya (chimpanzee) up 5 Mya (gibbon), demonstrating how challenging they are. We provide an evidence, that the UBCS statistic tracks a characteristic property of the human genomics, similar to the GC-content and consequently the BGC pattern [12, 13, 31] and, can provide more accurate dating. It should be also noted that the evolutionary distance of *Hylobatidae* and *Ponginae* from modern *Homo sapiens* are substantial, that predictions based only on one type of data become rather blurred and imprecise. A remedy to that could be to use multi-layer models that would bring together various types of genomic and other -omic data [32, 33].

More recently, mapping the sequenced reads from modern humans and ancient Hominini (French, Han, Papuan, San, Yoruba, *Neandertal*, *Denisovan*) to the chimpanzee reference sequence (pantro2 version) facilitated more precise speciation events dating [34]. Quality scores given by Burrows-Wheeler Aligner [35] and ANFO (https://bioinf.eva.mpg.de/anfo/) software packages for mapping low-divergent sequences against a large reference genome that aim to reflect the confidence of its mapping to the chimpanzee genome have been used. Further adequate thresholds and restrictions to filter out the tentative nucleotides were applied. For the remaining data, the total number of transversion substitutions between all possible pairs of organism samples was counted. Finally, correction of the genetic divergence for sequencing error was estimated and revealed two principal observations: (i) the pairwise comparison of divergence results between 7 *Hominins* suggest that *Neandertal* and *Denisovan* are on average genetically related to each other more than either of them is related to modern humans; (ii) assuming human-chimpanzee genetic divergence at 6.5 Mya *Neandertal* and *Denisovan* divergence from a common ancestor was estimated to 644,000 years ago, while the divergence of both *Neandertals* and *Denisovans* to present-day Africans was estimated to 812,000 years ago.

These results are consistent with the reports by Green et al. [36] who presented a draft sequence of the *Neandertal* genome. Using the numbers of transversions on the human lineage and the *Neandertal*-human ancestor to chimpanzee lineage the average divergence between DNA sequences in *Neandertals* and present-day humans, it was estimated as a percentage of the lineage from the modern human reference genome to the common ancestor of all considered organisms (i.e *Neandertals*, modern humans, and chimpanzees). The final estimate for the average divergence of *Neandertal* and modern human autosomal DNA sequences was estimated at 825,000 years ago, assuming the same human-chimpanzee split time.

## Conclusions and further research

Herein, we aimed to aggregate the available genomic knowledge about the Great Apes species in order to provide more accurate estimation of the HSA2 chromosomal fusion time. We used an improvement of the approach described by Dreszer et al. [11]. We point out the drawbacks of their UBCS statistic and propose the improvements that made it more robust to parameter changes as well as taking into account the cardinality of the repetitive weak-to-strong substitutions within the analyzed scope. Finally, we provide the time estimations of the major speciation events that have occurred on the human lineage.

A possible extension of the presented work is to analyze the *Hominini* genomes. We intend to estimate the speciation events of *Denisovans* and *Neandertals* based on the UBCS statistics. Another interesting task would be to use more sophisticated way to estimate the evolutionary distances among Great Apes utilizing UBCS statistics with an incorporation a formal statistical model. The aim would be to make use of the theory of Hidden Markov Models (e.g. as presented in [27, 37]) or to formulate a Bayesian, coalescent-based model, e.g. as the one by Gronau et al. [23].

## Methods

To better estimate the times of HSA2 fusion and split of modern human and Great Apes, we used the latest builds of these genomes. We present the derivation of the formulas used for the calculation of the UBCS statistics and emphasize the differences in calculations of the substitutions clusters as well as estimation method of the fusion time along with the determination of its confidence interval.

### Genomic data

All of the sequences and alignment files of the modern human and Great Apes genomes used in this study were downloaded from the UCSC Genome Browser (https://hgdownload.soe.ucsc.edu/downloads.html) [29]. Data used in this research are listed in the Additional file 1.

Data processing and analyses as well as statistical procedures were conducted using scripts written in the Python and R programming languages. The principal pipeline was implemented as a Snakemake [38] workflow to make it reproducible and scalable. All scripts and Snakemake workflow files are publicly available at GitHub Page: https://github.com/bposzewiecka/tytus.

### Identification of single-nucleotide differences between the modern human and Great Ape genomes

The analyses of the biased clustered substitutions (BCSs) require a distinction between the types of substitutions within the specific genomes.

First, single-nucleotide differences (SNDs) between the modern human and Great Apes genomes where identified using the reciprocal best alignments (the human genome was the target, and the Great Apes genomes were queries). The reciprocal best liftover chain file was used to map human genome regions to its homolog in the Rhesus (*Macaca mulatta*) genome.

Next, based on the processing procedures suggested by Dreszer et al. [11] (see also Additional file 1), SNDs between the modern human and Great Apes’ genomes were filtered. An SND was discarded if one of the conditions in the 11-base pair (bp) window with the SND in the middle was met: (i) a deletion or an insertion was present, (ii) more than 2 differences between the target and query were found, (iii) the target sequence could not be lifted-over to the Rhesus (*Macaca mulatta*).

Finally, each resulting SND was classified into one of the following three groups: (i) derived in target, (ii) derived in query, or (iii) inconclusive. If the human and Rhesus genome nucleotides were the same, the SND was classified as derived in query. Conversely, if the Great Apes and Rhesus genome nucleotides were the same, it was considered as derived in target. Other substitutions were classified as inconclusive. If the Rhesus base was A or T and derived base was C or G, the SND was considered as a *biased substitution*.

Having prepared the classification of SND between genomes, we proceed with their clustering and calculation of the statistics that summarizes the local enrichment in *biased substitutions*. Below, we refer to SND as a *substitution*.

**Fig. 5:**
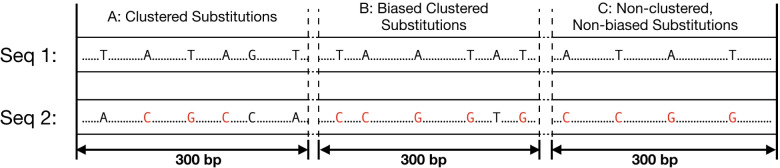
Examples of the substitutions classification for UBCS. The above figure depicts three 300 bp windows of two sequences, Seq 1 (reference) and Seq 2. Within each window substitutions that occurred on Seq 2 with respect to Seq 1 are denoted. The red color of the font is used for weak-to-strong substitutions. In the window A, all substitutions are considered as clustered substitutions (CSs), but not Biased Clustered Substitutions (BCSc), since only 50% of all substitutions are weak-to-strong. In the window B all substitutions are BCSs, because 5 out of 6 ($\ge$ 80%) substitutions are weak-to-strong. The remaining substitutions from the window C are neither clustered nor biased, because there are four substitution within this window

Dreszer et al. [11] defined the UBCS statistics as the difference between the observed and the expected number of BCSs in each window of 1 Mb (referred to as a region) on an entire chromosome (all windows are disjoint). For this purpose, a substitution is considered to be a *clustered substitution* (CS) if it belongs to a 300 bp window with at least four other substitutions. Next, a CS is considered a BCS if it belongs to a window with at least 80% of weak-to-strong substitutions (Fig. 5). In this setting, the null-hypothesis assumes no relationship between the bias towards weak-to-strong substitutions and the clustering of substitutions.

Nonetheless, Dreszer et al. [11] presented the method of computing the expected number of BCSs only for a simplified case when one substitution can be included in at most 2 clusters. However, especially in the subtelomeric regions containing GC-rich isochores [39], the structure of the intersecting clusters can be more complex.

More precisely, Dreszer et al. [11] relaxed the definition of CS by considering 300bp windows that start at coordinates that are multiples of 150. In such a case, the computation of the expected number of BCSs simplifies, as at most 2 clusters sharing the same substitution have to be considered. Dreszer et al. provided an example of computation the probability that a substitution is BCS in one specific arrangement of substitutions in the overlapping bins. The method is based on the conditioning on the number of substitutions in the first cluster. However, in our opinion, there are some inconsistencies in the derivations of these formulas broadly discussed in the Additional file 1. Dreszer et al. do not provide any estimates of the complexity of their method.

Here, we have devised an efficient algorithm allowing for the computation of the UBCS statistics considering windows starting at coordinates that are divisors of the window’s length. If a divisor is equal to 1, the algorithm during the computation of the probability that a given substitution is BC takes into account every possible window that the considered substitution is contained in. Such a procedure results in the precise calculation of the UBCS statistics by taking into account all possible window configurations of CSs. We also provide an estimation of the time and memory complexity of the described algorithm.

### Efficient algorithm for the calculation of the expected number of BCSs

The expected number of BCS can be obtained by summing the probability of being BC for each substitution in the genomic region. Here we present an algorithm for computing the probability that a substitution is biased clustered (BC) given $\hat{p}$ and the arrangement of substitutions in all windows containing it. In the calculation of the expected number of BCSs no association between bias and clustering is assumed. This quantity depends on the proportion of BS to all substitutions ($\hat{p}$) and the arrangement of substitutions in a genomic region.

Our algorithm compresses the genomic region containing each substitution into bins. Dynamic programming techniques allow for the computation of a probability that is tractable for the analyzed data in terms of time and computational memory consumption. To explain how the algorithm works, firstly we describe the procedure of compression of the genomic region containing the substitution in question into the vector of bins. Secondly, we present the derivations of formulas allowing for the application of the dynamic programming technique. Then, the pseudocode of the algorithm is shown. Finally, we explore the time and memory complexity of the algorithm.

#### The procedure of compression of the genomic region containing substitution into a vector of bins

Let us denote *W* as an event that a substitution at the coordinate *j* in the genome is BC and the respective probability as $p_{j}'$. To determine the value of $p_{j}'$ all windows containing this substitution have to be considered as the potential biased clusters. Let *m* be a size of a window, and $W_{i}$ an event that a window starting at a position $j-m+i$ is BC, where $i \in \{1, \dots , m\}$. The event *W* is a sum of the events that each window containing the coordinate *j* is BC, and can be expressed as:

$$\begin{aligned} p'_{j} = P(W) = P(W_{1} \cup W_{2} \cup \dots \cup W_{m}) \end{aligned}$$

**Fig. 6:**
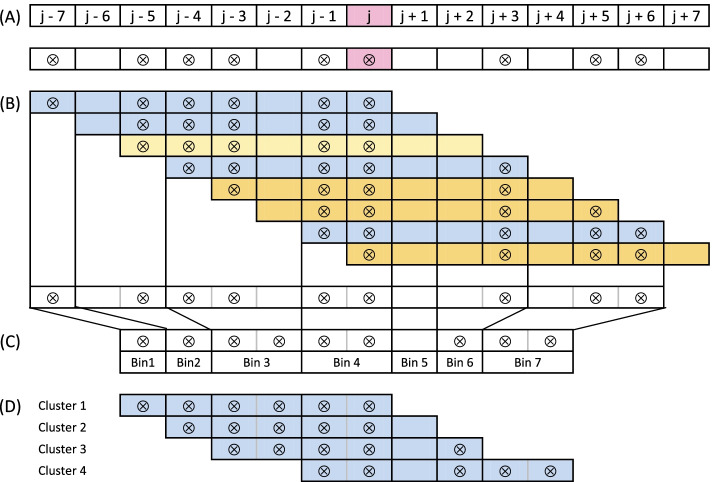
Illustration of the procedure of compression the genomic region containing a substitution into a vector of bins. The figure shows a compression procedure of a region of the genome containing a coordinate *j* and all windows of size $m = 8$ containing this coordinate. Substitutions are depicted as dots. **A** Configuration of substitutions around the considered substitution at the j-th coordinate in the genome. All windows containing substitution from j-th coordinate (depicted in pink) cover a region from $j -7$ to $j + 7$. **B** Classification of all possible windows containing substitution at the j-th coordinate. All windows of size *m* containing the j-th coordinate are shown. Representative windows are depicted in blue. Window depicted in yellow is excluded because it contains the same set of substitutions as the preceding window. Windows starting depicted in orange are excluded as they contain less than 5 substitutions. **C** Definition of bins based on all representative windows composition around the j-th coordinate. Starts and ends of representative windows (depicted by thick borders) mark the starts and ends of the bins. Note that substitution from the j-th coordinate is located in the middle bin. **D** Resulting set of clusters corresponding to the representative windows. These clusters will be used for calculation of the probability that substitution from the j-th coordinate is the BCS (see Algorithm 3)

The number of the components of the sum needed to compute $p'_{j}$ can be significantly reduced by unifying equal events and eliminating events with a zero probability. Therefore, from the windows that contain the same set of substitutions, only one representative can be left as a witness of being clustered. Windows containing less than 5 substitutions can be omitted, as their respective probabilities are zeros (because they are non-clustered).

Let us refer to the minimal set of windows that have to be considered in computing the probability of *W* after applying those rules as representative windows, and the number of such windows as *n*.

For the computation of a *P*(*W*), a region covered by the representative windows can be compressed to a vector of size $2 \cdot n - 1$. Each element of such a vector represents a fragment of this region and stores the number of substitutions contained within it. Let us refer to each element of a such compressed representation as a *bin*. The coordinates of starts and ends of consecutive *bins* are determined by the ordered coordinates of starts and ends of all representative windows. The *i*-th cluster is defined as *n* *bins* starting at the *i*-th position and corresponds to the *i*-th representative window. This procedure of the compression windows into bins ensures that the substitution from the *j*-th coordinate is contained in the middle *bin*.

The described procedure is outlined in the Fig. 6. The details of the method of selecting a minimal set of $W_{i}$ that is equivalent to *W* is described in the Additional file 1. We also prove that that the upper bound for the cardinality of this set is equal to the number of substitutions in the considered region.

#### Derivation of formulas used in the algorithm

The probability that s substitution form bin *n* is BC is equal to the probability of the sum of events that each cluster containing it is biased.

Let us denote:

- *A* as an event, that a substitution from *n*-th bin is BC (this substitution corresponds to the substitution located at *j*-th coordinate in the considered genomic region),
- $A_{k}$ as an event, that the *k*-th cluster is biased,
- $b_{k}$ as the number of substitutions in the *k*-th bin,

$$\begin{aligned} p'_{j} =P(A) = P(A_{1} \cup A_{2} \cup \dots \cup A_{n}) \end{aligned}$$

Notice that the event *A* corresponds to the previously considered event *W* and the selected representative windows $W_{i}$ correspond to the clusters $A_{i}$ and obviously $P(W)=P(A)$ . Now, the formula for *P*(*A*) can be written as a sum:

1 $$\begin{aligned}{}&P(A) = P(A_{1}) + P(A_{2} \cap {\neg} A_{1} ) + P(A_{3} \cap {\neg} A_{1} \cap {\neg} A_{2}) + \dots \\&+ \dots + P(A_{n} \cap {\neg} A_{1} \cap {\neg} A_{2} \cap \dots \cap {\neg} A_{n-1} ) \end{aligned}$$

According to the law of total probability, for each *k*, every component of the above sum (1) of a form $P(A_{k} \cap \lnot A_{1} \cap \lnot A_{2} \cap \dots \cap \lnot A_{k-1})$ can be expressed as:

2 $$\begin{aligned}{}&\sum {P(A_{k} \cap \lnot A_{1} \cap \lnot \cap \dots \cap \lnot A_{k-1} | X_{k} = x_{k}, \dots , X_{k+n-2} = x_{k+n-2} )} \; \cdot \\&\quad \quad P(X_{k} = x_{k}, \dots , X_{k+n-2} = x_{k+n-2})\end{aligned}$$

where $X_{k}$ is a random variable specifying the number of the biased substitutions in the k-th bin, the summation is done for all $x_{k} \in \{0, b_{k}\}, \dots , x_{k+n-2} \in \{0, b_{k+n-2}\}$. Next, since both events $A_{k}$ and $\lnot A_{1} \cap \lnot A_{2} \cap \dots \cap \lnot A_{k-1}$ are conditionally independent given $X_{k} = x_{k}, \dots , X_{k+n-2} = x_{k+n-2}$, each component of the sum (2) is equal to the product of the following three terms:

3 $$\begin{aligned}{}&P(A_{k} | X_{k} = x_{k}, \dots , X_{k+n-2} = x_{k+n-2} )\end{aligned}$$

4 $$\begin{aligned}{}&P( \lnot A_{1} \cap \lnot A_{2} \cap \dots \cap \lnot A_{k-1} | X_{k} = x_{k}, \dots , X_{k+n-2} = x_{k+n-2} ) \end{aligned}$$

5 $$\begin{aligned}{}&P(X_{k} = x_{k}, \dots , X_{k+n-2} = x_{k+n-2}) \end{aligned}$$

The first term (3) specifies the probability that a cluster is BC, given the frequencies of the first $n - 1$ bins. By the law of the total probability, it can be computed by conditioning on the frequency of the last bin in the cluster:

$$\begin{aligned}{}&P(A_{k} | X_{k} = x_{k}, \dots , X_{k+n-2} = x_{k+n-2}) \\&= \sum _{x_{k+n-1} \in \{ 0, s_{k+n-1} \}}{P(A_{k} | X_{k} = x_{k}, \dots , X_{k+n-1} = x_{k+n-1}) \cdot \; P(X_{k+n-1} = x_{k+n-1})} \end{aligned}$$

The value of the expression $P(A_{k} | X_{k} = x_{k}, \dots , X_{k+n-1} = x_{k+n-1})$ indicates that the *k*-th cluster containing $\sum _{i = k}^{k+n-1} x_{i}$ biased substitutions is biased.

The second term (4) specifies the probability, that the first $k - 1$ clusters are not biased, given the frequencies of the last $n - 1$ bins of the $k-1$-th cluster. By conditioning on the frequencies of the $k-1$-th bin, this probability can be expressed using the low of total probability as:

$$\begin{aligned}{}&P(\lnot A_{1} \cap \dots \cap \lnot A_{k-1} | X_{k} = x_{k}, \dots , X_{k+n-2} = x_{k+n-2} ) \\&= \sum _{x_{k-1} \in \{0, b_{k-1} \}} P( \lnot A_{1} \cap \dots \cap \lnot A_{k-1} |X_{k-1} = x_{k-1}, \dots , X_{k+n-2} = x_{k+n-2} ) \; \cdot \\&P(X_{k-1} = x_{k-1}) \end{aligned}$$

Yet events, $\lnot A_{1} \cap \dots \cap \lnot A_{k-2}$ and $\lnot A_{k-1}$ are conditionally independent given $X_{k-1} = x_{k-1}, \dots , X_{k+n-2} = x_{k+n-2}$, thus:

$$\begin{aligned}{}&P( \lnot A_{1} \cap \dots \cap \lnot A_{k-1} | X_{k} = x_{k}, \dots , X_{k+n-2} = x_{k+n-2} ) \\&= \sum _{x_{k-1} \in \{0, b_{k-1} \}} P(\lnot A_{k-1} |X_{k-1} = x_{k-1}, \dots , X_{k+n-2} = x_{k + n - 2}) \; \cdot \\&P( \lnot A_{1} \cap \dots \cap \lnot A_{k-2} |X_{k-1} = x_{k-1}, \dots , X_{k+n-2} = x_{k+n-2} ) \; \cdot P(X_{k-1} = x_{k-1}) \end{aligned}$$

The value of the probability $P(\lnot A_{k-1} |X_{k-1} = x_{k-1}, X_{k} = x_{k}, \dots , X_{k+n-2} = x_{k+n-2})$ indicates that the $k - 1$-th cluster containing $\sum _{i = k-1}^{k+n-2} x_{i}$ biased substitutions is biased.

Finally, events $\lnot A_{1} \cap \lnot A_{2} \cap \dots \cap \lnot A_{k-2} | X_{k-1} = x_{k-1}, \dots , X_{k+n-3} = x_{k+n-3}$ and $X_{k+n-2} = x_{k+n-2}$ are independent, thus $P( \lnot A_{1} \cap \lnot A_{2} \cap \dots \cap \lnot A_{k-1} | X_{k+1} = x_{k+1}, \dots , X_{k+n-2} = x_{k+n-2})$ is equal to:

$$\begin{aligned} P( \lnot A_{1} \cap \lnot A_{2} \cap \dots \cap \lnot A_{k-1} |X_{k-1} = x_{k-1}, \dots , X_{k+n-3} = x_{k+n-3}) \end{aligned}$$

#### Pseudocode of the algorithm

An algorithm for computing the probability that a substitution is BC is a straightforward application of the formulas derived above.

Computing all conditional probabilities given by the expression in Eq. (2) requires generating a Cartesian product representing all possible frequencies of biased substitutions in $n - 1$ subsequent bins. A pseudocode of the recursive function Generate-bin-frequencies is presented as Algorithm 1. This function returns a list of 2-tuples containing a list of frequencies together with their respective probabilities. 
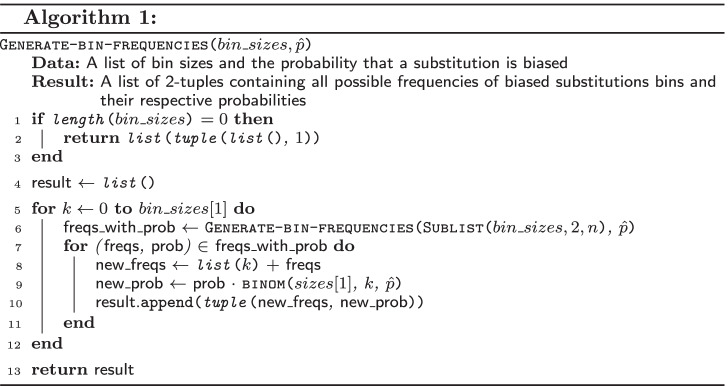


The function Binom-from($bin\_size$, $start\_size$, $\hat{p}$) (Algorithm 2) returns the probability that a bin of size $bin\_size$ contains $start\_size$ or more biased substitutions, where $\hat{p}$ is the probability that substitution is biased. 
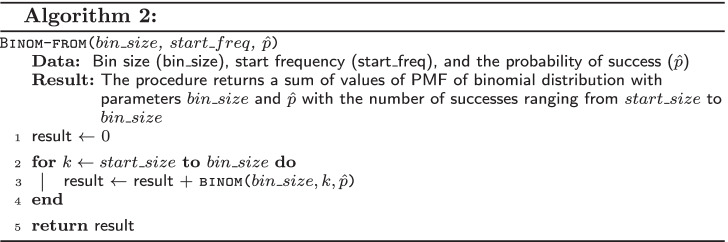


The function Get-probability-of-BCS($bin\_sizes$, $\hat{p}$) (Algorithm 3) takes as the arguments a list of sizes of consecutive bins of all clusters that contain a given substitution and a probability that the substitution is biased. The function returns the probability that the substitution contained in the middle bin is BC.

In the lines 4-5 of the Algorithm 3, the first component of the sum (1) is computed. Next components are evaluated in $n - 1$ iterations of the main loop in which the function Generate-bin-frequencies is used for generation of all possible frequencies of the BSs in subsequent $n - 1$ bins starting from the *k*-th bin.

Then, in the lines 16-18, the value of the conditional probability of the event that the cluster *k* is biased (term from Eq. (3)) is computed. In the lines 19-23, the value of the conditional probability that all previous clusters are not biased (term from Eq. (4)) is evaluated. For this purpose, the values from the dictionary $prev\_mem\_dict$ are used. The values in the dictionary $mem\_dict$ are updated for the use in the next iteration.

In the line 25, the result is updated by adding the product of the two probabilities (Eq. (3) and Eq. (4)) and the probability that $n-1$ bins contain the certain number of biased substitutions. 
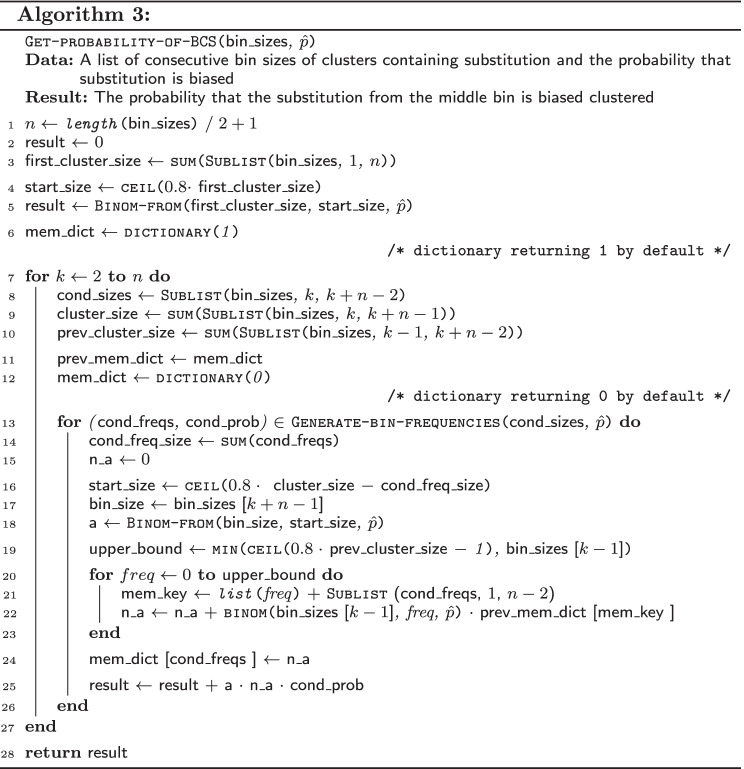


#### Analysis of the computational complexity of the algorithm

For the computation of the probability that a substitution is BC, the required memory is proportional to $2^{c}$, where *c* is the maximum number of substitution in a clusters. The inner loop iterates over Cartesian product representing all possible frequencies of the biased substitutions in $n - 1$ subsequent bins adding to the dictionary one value per iteration. Time complexity of the algorithm is proportional $n \cdot 2^{c}$ as main loop $n-1$ times iterate over Cartesian product defined above.

### UBCS based evolutionary distance estimation

Finally, to determine whether and how the average proportion between the values of the introduced UBCS statistics for two genome sequences within both telomere regions correlates with the time of evolutionary speciation events, we derived the following UBCS proportion measure.

Specifically, let us assume that there are two genome sequences $\mathcal {G}_{x}$ and $\mathcal {G}_{y}$, *N* chromosomes and *M* windows of size 1 Mb on telomeric regions of each chromosome. We denote $\mathcal {G}_{x_{j}^{i}}$ as *j*-th window on the *i*-th chromosome of the genome $\mathcal {G}_{x}$ and the value of its UBCS statistic as $\mathcal {U}(\mathcal {G}_{x_{j}^{i}})$, and $\overline{x}$ as the inverted sequence of *x* (i.e. the first window of $\overline{x}$ are the last 1 Mb of *x*). We calculate the average UBCS proportion between telomeres on *p* and *q* arms of *i*-th chromosomes of genomes $\mathcal {G}_{x}$ and $\mathcal {G}_{y}$ as:

$$\begin{aligned} \mathcal {T}_{p}(i) = \frac{ \sum _{j=1}^{M} \mathcal {U}(\mathcal {G}_{x_{j}^{i}}) }{{\sum _{j=1}^{M}\mathcal {U}}(\mathcal {G}_{y_{j}^{i}})} \qquad \mathcal {T}_{q}(i) = \frac{ \sum _{j=1}^{M}\mathcal {U}(\mathcal {G}_{\overline{x}_{j}^{i}}) }{ \sum _{j=1}^{M}\mathcal {U}(\mathcal {G}_{\overline{y}_{j}^{i}})} \end{aligned}$$

and the evolutionary distance based on the average UBCS proportion between genomes $\mathcal {G}_{x}$ and $\mathcal {G}_{y}$ as:

$$\begin{aligned} \mathcal {G}_{x} \vert \vert \mathcal {G}_{y} = \text {median}( \{\mathcal {T}_{p}(i): i \in {\mathcal{CT}\mathcal{}}_{p} \} \cup \{\mathcal {T}_{q}(i): i \in {\mathcal{CT}\mathcal{}}_{q} \} ) \end{aligned}$$

where ${\mathcal{CT}\mathcal{}}_{p}$ and ${\mathcal{CT}\mathcal{}}_{q}$ are sets of so called *control chromosomes* used to measure UBCS proportion between genomes on *p* and *q* arm respectively.

Such defined proportions allowed us to estimate the possible branching times in the evolutionary tree for each of the considered Great Apes genomes, that will be described in the next section.

For this purpose, we have computed the UBCS statistics using the human genome as a target and the Great Apes genomes as queries. Then, we have compared the distances between genome of the chimpanzee and all other Apes genomes by determining the value of an UBCS proportion $\mathcal {G}_{x} \vert \vert \mathcal {G}_{y}$ defined above. We have used 10 windows of the size of 1 Mb, and the following sets of the control chromosomes ${\mathcal{CT}\mathcal{}}_{p} = \{1, 4, 5, 6, 8, 10, 12, 16, 17, 19 \}$ and ${\mathcal{CT}\mathcal{}}_{q} = \{\text {all autosomes}\} \setminus \{15, 18, 19, 20\}$ for *p* and *q* arms, respectively. From the set of autosomal chromosomes, the short arms of the acrocentric chromosomes and the arms of chromosomes that were rearranged in human and Great Apes genomes were excluded. The confidence interval for the UBCS proportion was determined using the bootstrap method. The bootstrap sample was constructed by sampling with replacement of the 15 out of 28 telomeres, and 8 of 10 windows on the basis of which UBCS proportion is calculated. The sampling procedure was repeated 1000 times for each species, and confidence intervals were determined by eliminating 5% of the most extreme values. Speciation time was approximated by multipliaction the UBCS proportion (quantifying the distance between the chimpanzee genome to genome of interest) by the estimated time of the human-chimpanzee split. We have fixed the human-chimpanzee speciation time at 6 Mya. The confidence intervals for the speciation events were obtained by rescaling the confidence intervals of the UBCS proportions in the same manner. For the purpose of more informative visualizations, in all of the figures regarding UBCS statistics, the loess regression function was used to smooth the curves.

### UBCS based estimation of the fusion time

Our method of estimation of the HSA2 fusion time is based on the following key assumptions. First, analogously to Dreszer et al. [11], we assumed a constant evolutionary force that has lead to the accumulation of BCS near telomeres in each species. The second assumption considers the time of human-chimpanzee split at approximately 6 Mya. Finally, since the chimpanzee chromosomes 2A and 2B are capped with hyper-expanded segmental duplications and tandem repeats (StSats) not existing in the human genome [8, 40], we cannot measure the magnitude of the accumulation of BCS in those fragments comparing to the human reference. To account for this fact, the values derived in chimpanzee near the fusion site must be rescaled.

Note, that the method of the fusion time estimation proposed by Dreszer et al. [11] used also an additional assumption that the ratio of UBCS between the *p* and *q* arms of any chromosome is similar for human and chimp. This assumption is clearly violated in the data and therefore we have devised a different estimation procedure.

For the calculation of the fusion time, let us define *R* as a proportion of time of the last 6 Mya that two chromosomes were not fused. Then, the fusion time can be estimated as $6 \, \text {Mya} \cdot (1-R)$. We can approximate *R* as a ratio of two quantities: (i) the proportion of the UBCS values between derived in human and in the chimpanzee in the region next to the fusion site (homologous to the chimpanzee *p* arm on chromosome 2A and *p* arm on chromosome 2B) and (ii) the proportion of the UBCS values derived in human in the region at the beginning of the telomere and in the corresponding region of the same length located a few Mb away. The former proportion, reflects the decline in the accumulation of BCS after the fusion event, the latter accounts for the rescaling the signal of UBCS derived in the chimpanzee near the fusion site taking into account the existence of additional sequences at the beginning of telomeres of the chimpanzee chromosomes.

To estimate the rescaling factor, we have probed the control chromosome telomeres UBCS statistics derived in human in two intervals: first started at the beginning of the telomere and second at the fifth megabase from the telomere. For this estimation we have used the following sets of control chromosomes ${\mathcal{CT}\mathcal{}}_{p} = {\mathcal{CT}\mathcal{}}_{q} = \{1..12, 16, 17\}$.To increase the robustness of the procedure we repeated the calculation for the telomeric regions of different sizes (from 15 Mb to 20 Mb). The final evaluation of the fusion time used a median value of the proportions along with corresponding 95% confidence interval.

## Data Availability

All data used for this study were downloaded from the UCSC Genome Browser download page (https://hgdownload.soe.ucsc.edu/downloads.html) [29].

## Abbreviations

BC: Biased clustered
BCS: Biased clustered substitution
BGC: Biased gene conversion
CS: Clustered substitution
HSA2: Human chromosome 2
LCA: Last common ancestor
ML: Maximum likelihood
Mya: Million years ago
NGS: Next generation sequencing
SD: Segmental duplication
SND: Single-nucleotide difference
UBCS: Unexpected Bias Clustered Substitutions
